# Myocardial Tissue Characterization in Heart Failure with Preserved Ejection Fraction: From Histopathology and Cardiac Magnetic Resonance Findings to Therapeutic Targets

**DOI:** 10.3390/ijms22147650

**Published:** 2021-07-17

**Authors:** Paolo Severino, Andrea D’Amato, Silvia Prosperi, Francesca Fanisio, Lucia Ilaria Birtolo, Bettina Costi, Lucrezia Netti, Cristina Chimenti, Carlo Lavalle, Viviana Maestrini, Massimo Mancone, Francesco Fedele

**Affiliations:** Department of Clinical, Internal, Anesthesiology and Cardiovascular Sciences, Sapienza University of Rome, Viale del Policlinico, 155, 00161 Rome, Italy; paolo.severino@uniroma1.it (P.S.); damatoandrea92@gmail.com (A.D.); silviapro@outlook.it (S.P.); fanisio.francesca@gmail.com (F.F.); ilariabirtolo@gmail.com (L.I.B.); bettina.costi@gmail.com (B.C.); lucrezia.netti@gmail.com (L.N.); cristina.chimenti@uniroma1.it (C.C.); carlo.lavalle@uniroma1.it (C.L.); viviana.maestrini@uniroma1.it (V.M.); massimo.mancone@uniroma1.it (M.M.)

**Keywords:** heart failure, heart failure with preserved ejection fraction, myocardial tissue characterization, cardiac magnetic resonance, endomyocardial biopsy, therapy

## Abstract

Heart failure with preserved ejection fraction (HFpEF) is a complex clinical syndrome responsible for high mortality and morbidity rates. It has an ever growing social and economic impact and a deeper knowledge of molecular and pathophysiological basis is essential for the ideal management of HFpEF patients. The association between HFpEF and traditional cardiovascular risk factors is known. However, myocardial alterations, as well as pathophysiological mechanisms involved are not completely defined. Under the definition of HFpEF there is a wide spectrum of different myocardial structural alterations. Myocardial hypertrophy and fibrosis, coronary microvascular dysfunction, oxidative stress and inflammation are only some of the main pathological detectable processes. Furthermore, there is a lack of effective pharmacological targets to improve HFpEF patients’ outcomes and risk factors control is the primary and unique approach to treat those patients. Myocardial tissue characterization, through invasive and non-invasive techniques, such as endomyocardial biopsy and cardiac magnetic resonance respectively, may represent the starting point to understand the genetic, molecular and pathophysiological mechanisms underlying this complex syndrome. The correlation between histopathological findings and imaging aspects may be the future challenge for the earlier and large-scale HFpEF diagnosis, in order to plan a specific and effective treatment able to modify the disease’s natural course.

## 1. Introduction

Heart failure with preserved ejection fraction (HFpEF) is a complex and multifaceted clinical syndrome. HFpEF percentage is between 22% and 73% of all heart failure (HF) cases [1,2,3,4] and it is increasing, in contrast to heart failure with reduced ejection (HFrEF) [5]. HFpEF patients are often female, aged, obese, hypertensive and with atrial fibrillation history [6]. It is a syndrome containing an heterogenous disease population, characterized by patients with valvular disease, hypertensive cardiopathy, amyloidosis, genetic cardiomyopathies. This heterogeneity accounts for the several structural myocardial and coronary alterations seen in HFpEF, which are partially unknown yet [7,8,9]. After one year from diagnosis, mortality in HFpEF is about 20–29% [10] and, after five years, about 50% of HFpEF patients die [10,11]. Compared to HFpEF, all-cause mortality is higher in HFrEF, although its incidence is decreasing [10,12]. Furthermore, in-hospital mortality, although initially slightly more frequent in the HFrEF group, after 2–3 months become equivalent in the two HF groups [10,13]. 

In HFpEF, the underlying myocardial alterations are incompletely defined. Myocardial histopathological alterations have been characterized through invasive methods, such as endomyocardial biopsy, but also cardiac magnetic resonance (CMR) or other imaging technique are able to identify them in a simpler and non-invasive way. However, the correlation between histopathological findings and imaging aspects is still a challenge for medicine. Myocardial fibrosis, cardiomyocytes hypertrophy, coronary microvascular dysfunction (CMD) and inflammation have been identified as major pathological processes that affect HFpEF myocardium. Although these mechanisms have been also identified in HFrEF, several observations highlight that their spatial and temporal onset and progression may differ between the two conditions. HFpEF hearts appear remodeled towards a hypertrophic shape, and not dilated as HFrEF, left atrial dilation is very frequent and most of HFpEF patients have evidence of diastolic dysfunction. In this regard, the advanced imaging techniques, and in particular the refined tissue characterization allowed by CMR is a very valid tool [14]. For an initial evaluation of suspected HFpEF patient, echocardiography is still the imaging of choice [15], but most of the parameters are load-dependent and do not always allow to detect diastolic dysfunction and, above all, to distinguish the various underlying causes [16]. With regard to increase in filling pressures, which is an HFpEF characteristic element, the gold standard remains the invasive catheterization, which allows the calculation of the constant tau and beta, to describe the first and last part of diastole respectively [17]. CMR overcomes the limits of echocardiography, allowing not only to evaluate dimension, wall thickness and function, but especially to describe the specific tissue composition, such as the presence of focal fibrosis, edema, fat, iron overload [18]. Over the last decade the introduction of Mapping techniques improved the ability to characterize the tissue and quantify the diffuse processes [19]. Both native myocardial T1 and extracellular volume fraction (ECV) have a proven correlation with histopathological data [20,21,22,23]. ECV shows a significant association with increased myocardial type 1 collagen deposition [24]. Native myocardial T1 is a composite signal from intracellular and extracellular component, while ECV quantify the extracellular space. ECV expresses the extracellular space percentage, and it is obtained from the difference between T1 times, before and after gadolinium administration corrected by the hematocrit.

Myocardial tissue characterization is a crucial aspect for the comprehension of HFpEF molecular basis, as well as pathophysiological aspects. Currently, there is no specific therapy for HFpEF and risk factors control is the primary and unique approach for HFpEF management and prevention [25]. T1 Mapping technique has been demonstrated to increase the ability to characterize the several possible etiologies of HFpEF [26,27,28].

The aim of this review is to provide an overview about the most recent findings about myocardial tissue characterization in HFpEF, through invasive and non-invasive methods, in order to allow a wide comprehension of HFpEF pathophysiological mechanisms and provide new therapeutic targets for its treatment. 

## 2. The Pathophysiology of Heart Failure with Preserved Ejection Fraction 

HF is a complex syndrome characterized by the inability of heart to guarantee an adequate blood output to peripheral organs or to provide it through cardiac filling pressure increase. The definition of HFpEF is clinical and coupled with the evidence of a left ventricular ejection fraction >50% [9].

**Figure 1:**
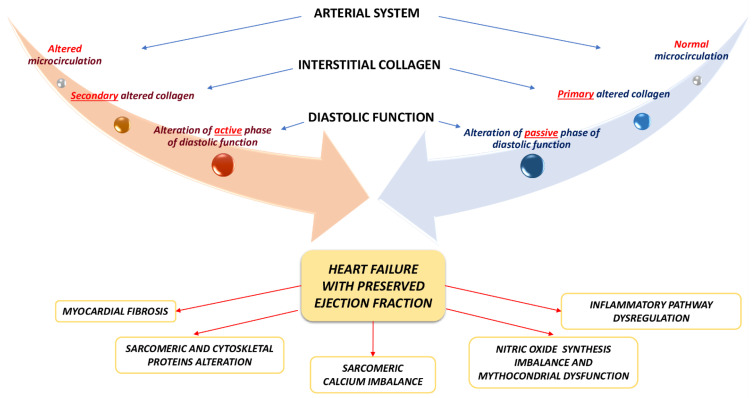
Possible pathophysiological pathways and molecular mechanisms involved in heart failure with preserved ejection fraction (HFpEF). Depending on the prevalence of microvascular dysfunction or excessive and abnormal collagen deposition, two main different pathophysiological HFpEF patterns could be outlined: (1) an HFpEF pattern with impaired passive phase of diastolic function, caused by altered quantity and quality of interstitial collagen, but with normal microcirculation (expressed by the blue arrow) and (2) an HFpEF pattern with impaired active phase of diastolic function, induced by structural and functional microvascular alteration, with secondary interstitium involvement (expressed by the orange arrow). In the lower part of the figure, the main molecular mechanisms observed in HFpEF have been illustrated.

Pathophysiology of HFpEF is less deciphered than HFrEF, since there are different underlying etiologies, pathways, phenotypes and comorbidities. From the pathophysiological point of view, in HFpEF, left ventricular concentric remodeling and/or hypertrophy have been observed [4,7,8]. HFpEF patients left ventricle shows high filling pressure and diastolic dysfunction. LV diastolic dysfunction is defined by the active phase of relaxation impairment, an increase in viscoelastic chamber stiffness (passive phase), or a combination of the two. Many histopathological mechanisms are the substrates of left ventricle stiffness, such as myocardial fibrosis, titin and other sarcomeric proteins alterations, sarcoplasmic calcium reuptake imbalance and cytoskeleton alterations during protodiastole (Figure 1). Three interconnected heart compartments are involved in HFpEF pathophysiology: coronary microcirculation, myocardium and extracellular space. Depending on the prevalence of microvascular dysfunction or excessive and abnormal collagen deposition, two main different HFpEF patterns could be outlined: (1) an HFpEF pattern with impaired passive phase of diastolic function, caused by altered quantity and quality of interstitial collagen, but with normal microcirculation and (2) an HFpEF pattern with impaired active phase of diastolic function, induced by structural and functional microvascular alteration, with secondary interstitium involvement (Figure 1). In HFpEF hearts, only an elevated left atrium- left ventricle gradient allows ventricular filling, and it associates with increased atrium pressure [4,7,8]. Increased left ventricular filling pressure, as well as, left atrium hypertension are associated with pulmonary hypertension and right ventricle dysfunction, which has a significant impact on prognosis and outcomes [10]. Consequently, left ventricle filling become more dependent on left atrium contraction, but progressive left atrium dysfunction and atrial fibrillation further worsen left ventricle filling and pulmonary hypertension. Right ventricle pressure overload and dysfunction determine systemic venous congestion and, therefore, renal impairment, oedema, hepatic and intestinal congestion. On the other hand, according to the interdependence ventricular principle, the right ventricle pressure overload and dysfunction worsen left ventricle filling, triggering a vicious circle which determines HFpEF progression [4,7,8]. In addition, systolic function is compromised in HFpEF, despite ejection fraction is normal and, in fact, ejection fraction is not always related with symptoms and clinical findings, in patients with HF [29,30,31,32,33,34]. HFpEF related systolic dysfunction is characterized by a prevalent left ventricular longitudinal shortening involvement, as confirmed by CMR feature tracking [35,36]. Systolic function is actually closely related to the diastolic, because during the systolic phase, the myocardium accumulates energy that allows a satisfying relaxation, during the diastolic phase [35]. Then, if the longitudinal systolic function is reduced, also diastolic dysfunction will be affected. Ito et al. confirmed the close relationship between the relaxation constant tau and the value of global longitudinal strain (GLS), allowing a reliable and realistic diastolic function estimation, through a systolic parameter [36]. Moreover, reduced stroke volume and cardiac output, as well as chronotropic incompetence have been also observed [4,7,8]. These findings mainly occur under stress condition. For this reason, HFpEF related systolic dysfunction is emphasized during physical exercise, remaining often normal at rest. Finally, cardiac output reduction is also due to ventricle-arterial coupling imbalance, determined by increased vascular stiffness and endothelial dysfunction [7,8]. 

### 2.1. Heart Failure with Preserved Ejection Fraction and Myocardial Morphological Alterations: Cellular and Interstitial Involvement

Myocardial fibrosis is characterized by extracellular matrix components storage. Myocardial fibrosis is a specific histopathological process related to myocardial remodeling and it is closely associated with HF. This histopathological mechanism underpins many genetic, epigenetic and molecular abnormalities, such as excessive extracellular matrix production, post-translational collagen cross linking abnormalities, as well as matrix metalloproteinases dysregulation [7,8,37]. Myocardial fibrosis extent is also influenced by gender and race differences and cardiovascular risk factors prevalence. In this regard, it is mainly seen in African American more than Caucasian and in female more than male gender, due to high prevalence of hypertension, kidney disease and diabetes in these population [38,39,40]. 

From the clinical point of view, the myocardial fibrosis spatial distribution and temporal onset may play a prognostic role, being involved in systolic and diastolic dysfunction, as well as arrythmias [37,38,39,40,41,42]. In HF patients, the myocardial fibrosis may vary in term of spread and entity, defining a personalized and specific fibrosis distribution pattern. For example, reparative fibrosis occurs after myocardial infarction and it constitutes the scar, which is localized in a myocardial region according with the occluded coronary artery. It has mechanic and conservative properties because fibrotic tissue stabilizes a *locus minoris resistentiae* developed in the infarct area. On the other hand, reactive fibrosis is more associated with non-ischemic myocardial involvement, in condition of pressure overload [37]. Specifically, in HFrEF myocardial fibrosis is characterized by reparative and localized fibrosis in scar area, while HFpEF is characterized by reactive, diffuse and perivascular fibrosis [43,44,45]. Reparative fibrosis is characterized by cardiomyocytes replacement, as it happens in myocardial infarction, while the reactive fibrosis is characterized by increased interstitium collagen deposition [46]. Both focal and diffuse fibrosis are present in HFpEF, but the latter is prevalent. Focal fibrosis, both ischemic and non-ischemic, is well visualized by the late gadolinium enhancement (LGE) technique [19] Specifically, in HFpEF patients, Kanagala et al. identified that half population had LGE with the non-ischemic being the prevalent form [47]. Not only the diffuse fibrosis degree, calculated by ECV, but also the collagen and the crosslinking type affect the diastolic dysfunction [24,48]. Su et al. found higher ECV in HFrEF than in HFpEF group [46]. However, in HFrEF, ECV was not related to ventricular dysfunction because the main HF cause was a scarring fibrosis. 

Considering the hemodynamic and clinical impact of myocardial fibrosis, several aspects should be related with the increasing left ventricular filling pressure, worse diastolic function and hospitalization, in HFpEF patients. In this regard, type 1 collagen production instead of type III collagen, a raise in collagen storage and cross linking have been observed [43,49,50,51]. In particular, collagen cross linking may be induced by advanced glycation products (AGEs) and lysyl oxidases overexpression, in patients with type 2 diabetes mellitus (T2DM) and diastolic dysfunction. In those patients, collagen quantity and cross linking related with diastolic dysfunction parameters identified through Tissue Doppler, at transthoracic echocardiography [43,49,52]. In case of pressure overload, extracellular matrix and collagen myocardial deposition have been observed. In this mechanism, the transforming growth factor beta (TGF-β) plays a crucial role. Myocardial strain, cellular stress, as well as endothelin-1 and angiotensin II are TGF-β activation promoters. TGF-β pathway is associated with p38, Ras homologous/Rho-associated protein kinase (Rho/ROCK), Smad 2/3, Galectin-3 and extracellular matrix grow factors activation. Moreover, several fibroblasts activation markers have been identified, such as fibroblasts activating protein (FAP) and Periostin [53]. ECV has a strong correlation with the collagen percentage found in endomyocardial biopsies [24,54] The extracellular component rise and fibrosis are the main cause of myocardial stiffness, detected by beta constant increase, as demonstrated by Rommel et al. [55]. 

Cardiac amyloidosis is one of the causes for HFpEF and its prevalence, in this population, is constantly increasing [56]. Transthyretin cardiac amyloidosis is characterized by extracellular insoluble transthyretin amyloid deposition [56]. Two types of insoluble transthyretin, variant or wild type, may constitute amyloid fibrils. Through non-invasive approaches, the prevalence of transthyretin amyloidosis in HFpEF patients is 13% [57]. Hahn et al. reported a prevalence of cardiac amyloidosis around 14% in a cohort of HFpEF patients and 50% of them could not be identified by screening criteria, suggesting that cardiac amyloidosis diagnosis can be underdiagnosed in patients with HFpEF [38]. Several clinical features were identified in patients with HFpEF and cardiac amyloidosis, such as lower body mass index (BMI), lower arterial blood pressure values, advanced age, high Troponin-I and N-terminal pro b-type natriuretic peptide (NT-proBNP) values, as well as raised left ventricular wall thickness [38]. However, the real prevalence of transthyretin amyloidosis should be higher, considering that about 25% of patients aged more than 80 years show myocardial wild-type transthyretin fibrils in autopsy studies [57,58]. The high prevalence of occult cardiac amyloid in general population was evaluated by Treibel et al. who demonstrated the presence of cardiac amyloid, in 6% of patients with more than 65 years who underwent surgery because of aortic stenosis. Moreover, cardiac amyloid presence in these patients was associated with worse prognosis [59]. CMR gave a great contribution allowing the identification of cardiac amyloid deposition through typical images with abnormal gadolinium kinetics, global subendocardial LGE characteristic distribution, increased ECV and high native T1 values [57,60]. The CMR pathological findings match with heart walls amyloid deposition found on biopsy [60]. Moreover, the transmural LGE pattern has also a determining role in distinguishing the transthyretin amyloid type, with a better prognosis, from the immunoglobulin light-chain amyloid type [61]. 

Reduced left ventricular compliance and relaxation are mainly due to myocardial fibrosis and increased myocardial stiffness induced titin post-translational modifications. Titin is a big size sarcomeric protein involved in myocardial stiffness. Titin is produced in two main isoforms: the longer N2BA and the shorter N2B. In HFpEF patients, the N2BA/N2B ratio is altered. Oxidative stress and reduced nitric oxide (NO) production, are associated with impaired nitric oxide/cyclic guanosine monophosphate/protein kinase G (NO/cGMP/PKG) pathway and N2B titin post-translational modification [51,62]. Moreover, an increased N2B isoform expression has been observed and it associates with increased myocardial stiffness [51,62,63]. 

Microtubules have a main role in the cardiomyocytes and myocardial compliance. In failing heart, microtubules are detyrosinated and mechanically uncoupled, determining increased viscoelastic resistance to diastolic stretch [64]. The role of microtubules in the myocardial compliance regulation is reduced at increased cardiomyocytes stretching [64]. In this regard, the role of extracellular matrix and proteins became predominant. For this reason, microtubules may represent a therapeutic target, in order to preserve myocardial compliance through the diastolic associated viscous forces reduction [64]. Moreover, many cardiomyocytes intracellular alterations, such as actin filaments fragmentation and nuclear morphological alterations, have been identified in diabetes mellitus and other comorbidities often seen in patients with HFpEF. In this regard, cytoskeletal disorganization represents a main mechanism involved in myocardial stiffness, during diastolic dysfunction occurring in diabetic cardiomyopathy [53,64,65,66]. The cardiomyocytes sarcoplasmic reticulum calcium-ATPase 2 (SERCA2) expression is markedly reduced, due to reactive oxygen species (ROS) and AGEs storage. Consequently, intracellular calcium values remain high. This alteration leads to two main conditions: first, it determines a reduced cardiomyocytes relaxation and increased stiffness, secondly a sarcoplasmic calcium reserves reduction, which causes impaired cardiomyocytes contraction and reduced global myocardial contractility [50,65,66] Other cardiomyocytes alterations have been described in HFpEF. Increased cardiomyocytes dimensions are associated with lower cGMP and PKG activity participating to the concentric hypertrophy progression seen HFpEF. On the other hand, in HFrEF cardiomyocytes are more stretched and thinner and they are characterized by less passive stiffness and myofibrillar density [56,65,66]. The left ventricle geometric remodeling contributes to the filling pressures increase [67]. Different studies have shown an association between ventricular hypertrophy and constant tau of relaxation [68]. An explanation for this process could be that the cardiomyocyte size increase is not counterbalanced by an adequate increase in perfusion [69]. Moreover, the cardiomyocytes mitochondrial dysfunction causes inadequate myocardial relaxation [70]. 

There is a relationship between CMR parameters, such as native myocardial T1 and ECV, and invasive parameters [55]. Omori et al. performed a study including CMR with native Myocardial T1 and ECV, invasive evaluation of filling pressures and endomyocardial biopsy, in HFpEF patients [71]. They found that native T1 and especially ECV were higher in HFpEF than in the control group and that they are both reliable in determining the percentage of collagen volume fraction and the passive stiffness of the left ventricle, calculated invasively through the beta relaxation constant [71]. ECV has a proven validation with the histopathology found through intraoperative biopsy studies performed at the time of aortic stenosis or heart transplant surgery [62,72,73].

The CMR importance in HFpEF also lies in its ability to predict patients’ prognosis and outcome. Poyhonen et al. found that HFpEF patients with CMR positive for LGE showed an increased risk of adverse events, including death [74]. Kato et al. demonstrated a sensitivity of 80% and a specificity of 77% for LGE in predicting poor prognosis in HFpEF patients [75]. Mascherbauer et al. found that T1 mapping abnormal value was associated with death and/or re-hospitalization, in HFpEF patients [76]. T1 mapping value is associated with extracellular matrix increase and worse prognosis in HFpEF [77]. Recently, Schelbert et al. demonstrated the ECV ability to predict mortality [78]. In this regard, ECV may have an important prognostic role as increased mortality and morbidity indicator [79]. In fact, HFpEF patients with ECV above normal values showed increased cardiovascular event risk. This prognostic correlation was higher in the first six months of follow-up [80]. 

### 2.2. Heart Failure with Preserved Ejection Fraction and Coronary Microvascular Dysfunction: Morphological and Functional Aspects

CMD is a condition characterized by coronary flow reserve (CFR) impairment in absence of coronary epicardial obstruction or unaltered flow fractional reserve (FFR) [81,82,83,84,85]. Endothelium-independent involvement after intracoronary adenosine administration is defined by a CFR value lower than 2.5–2.0, increased microvascular resistance index (IMR) upper 25, FFR > 0.8 and hyperemic myocardial velocity resistance ≥1.9 [81,82]. Endothelium-dependent involvement is defined by vasoreactivity test with sequential acetylcholine administration [81,82]. CMD represents a pivotal chronic coronary syndrome pathophysiological substrate, determining ischemia with non-obstructive coronary artery disease (INOCA) [81,82,83,84,85]. However, it has been also associated with acute coronary syndromes, being the cause of myocardial infarction with non-obstructive coronary artery disease (MINOCA) [81,82,83,84,85]. Many other myocardial conditions associate with CMD, such as inflammation, as occurs in myocarditis, hypertrophy and myocardial storage disorders, as occurs in hypertrophic and infiltrative cardiomyopathy. In the setting of CMD and myocardial ischemia, several microvascular functional and morphological alterations have been involved, beyond the classical mechanisms [81,82,83,84,85,86,87,88]. Endothelial dysfunction is a key mechanism in CMD. Endothelial dysfunction is sustained by oxidative stress, NO synthesis imbalance, increased response to endothelin, mitochondrial dysfunction, impaired endothelial cells repair [84]. Endothelial dysfunction is associated with reduced estrogens production and estradiol administration may improve diastolic function in post-menopausal women [84,89]. Renin-angiotensin aldosterone system (RAAS) hyperactivation promotes the microvascular resistances increase, as well as sympathetic hyperactivity and alfa-adrenergic tone prevalence, in patients with CMD [81,82,83,84,85,86,87,88]. In addition, endothelium independent vasodilation imbalance, characterized by excessive smooth muscle vasoconstrictor tone, has been described in HFpEF patients as CMD mechanism [81,82,83,84,85,86,87,88]. Among morphological alterations, microvascular rarefaction and hyperpermeability and inward hypertrophic remodeling have been described as main CMD histopathological alterations [81,82,83,84,85,86,87,88]. CMD has been described in association with HF, in particular HFpEF related risk factors, such as diabetes mellitus, dyslipidemia, ageing, arterial hypertension, female gender and obesity [81,82,83,84,85,86,87,88,89,90,91,92,93]. Arterial hypertension and RAAS hyperactivation promote microvascular inward hypertrophic remodeling, inducing myocardial ischemia, HF and chronic kidney disease, which is associated with HFpEF. Dyslipidemia determines microvascular endothelial dependent vasodilation impairment. This is mediated by oxidative stress and inflammation response activation through oxidized LDL (Ox-LDL). Obesity is characterized by epicardial and coronary perivascular adipose tissue storage and inflammation. In diabetes mellitus the relationship with CMD is bidirectional and a main role of oxidative stress and hyperglycemia has been described [81,82,83,84,85,86,87,88,89,90,91,92]. 

As previously discussed, HFpEF related CMD is sustained by microcirculation histopathological alterations [43]. Microvascular rarefaction is a frequent finding in patients with HFpEF and it promotes reduced myocardial perfusion and increased cardiomyocytes oxygen extraction, leading to myocardial metabolic impairment [43,94]. Moreover, microvascular rarefaction is perpetuated by cardiovascular comorbidities often seen in HFpEF patients, such as hypertension, diabetes mellitus and aging. Coronary microvascular rarefaction is associated with increased left ventricular filling pressure, subepicardial adipose tissue storage, as well as myocardial oedema and fibrosis [43,95]. Moreover, reduced microvascular density worsens cardiovascular comorbidities such as diabetes, due to impaired insulin tissue delivery, and hypertension [43,96]. In diabetes, microvascular rarefaction is determined by several pathophysiological mechanisms, such as angiostatic proteins accumulation, endothelial cells apoptosis and pericytes rarefaction [97,98]. Moreover, microvascular rarefaction is diffuse and proportionate along the different myocardial region. In those patients, myocardial fibrosis and microvascular rarefaction were inversely related. This observation supports a pathophysiological role of microvascular endothelial inflammation and dysfunction in the microvascular rarefaction, myocardial fibrosis and hemodynamic alterations seen in HFpEF [50]. Microvascular rarefaction determines CFR impairment, which is associated with reduced myocardial perfusion and cardiomyocytes death, due to ischemia. In diabetes mellitus, coronary microvascular vasodilatory response is reduced, due to endothelial dysfunction and NO reduced bioavailability. In this regard, endothelial dysfunction is due to hyperglycemia, which promotes vessels local inflammation, leucocytes migration and ROS production. Hyperglycemia is also associated with sympathetic and smooth muscle cells dysfunction, as well as increased alfa-adrenergic tone. 

Another important morphological alteration is microvascular hyperpermeability that has been observed in HFpEF related comorbidities, as well as in HFrEF and ischemia-reperfusion injury. Several molecular components are involved in the microvascular wall dysfunction. In this regard, the glycocalyx alteration promotes the adhesion molecules formation and neutrophils migration [43,99]. Pro-inflammatory molecules induce microvascular hyperpermeability through intercellular junctions’ loss. It is characterized by vascular endothelial cadherin hyperphosphorylation and trans-endothelial channels formation from vesciculo-vacuolar organelles [43,100]. Pericytes alterations and distribution are associated with microvascular tone and blood flow dysregulation, in HFpEF [43,101]. Zeng et al. found that impaired HIF-2α/Notch3 and angiopoietins/Tie-2 pathways may bring to pericytes loss and reduced endothelial cells coverage, as well as pericytes-myofibroblasts transition. The latter is associated with increased myocardial fibrosis and stiffness [102,103]. In a study based on autopsies, Mohammed et al. found that microvascular density was lower, and fibrosis was more severe in patients with HFpEF [50]. CMD seen during myocardial ischemia and HF may be also determined by coronary ion channels impairment [90,91,92,104,105,106,107]. With this regard, endothelial and smooth muscle cells coronary ion channels mediate, form the molecular point of view, the coronary blood flow regulation mechanisms physiological effects [90,91,92,104,105,106,107]. For this reason, their impairment determined by ROS, AGEs, inflammation, increased shear stress and genetic predisposition affects the coronary vascular tone regulation [90,91,92,104,105,106,107]. These aspects promote microvascular rarefaction, as well as myocardial ischemia, fibrosis and, therefore, HF. 

The lymphatic vascular system may be involved in the pathophysiology of HFpEF, contributing to the different myocardial histopathological findings observed in this condition [43]. In particular, lymphatic alterations may contribute to diastolic dysfunction, in HFpEF. Lymphatic dysregulation and obstruction are a cause of impaired left ventricular compliance and contractility. Moreover, it associates with widespread and lung interstitial oedema according to HFpEF progression [43,108,109]. Several cardiovascular risk factors associated with HFpEF are characterized by lymphatic network remodeling [43,110,111]. Hypercholesterolemia and high BMI values are associated with lymphatic network rarefaction. Hypertension is associated with pro-lymphangiogenesis molecules production and lymphatic transportation imbalance [43,112,113,114]. In this regard, several molecules inducing lymphangiogenesis, such as vascular endothelial growth factor (VEGF) -C and -D may represent new treatment strategies against HFpEF [43,115]. Although coronary involvement in HFpEF is poorly described in literature, coronary artery disease (CAD) more diffuse and extensive, involving more vessels, in HFpEF has been described. CAD extension and severity may be inversely related with ejection fraction [50]. 

Kato et al. study was the first to demonstrate the CFR impairment in HFpEF. They used phase contrast cine-magnetic resonance imaging for a non-invasively calculation of CFR, obtained from the ratio of coronary sinus blood flow with and without adenosine triphosphate (ATP) infusion [116]. Results that confirmed a CFR reduction in 76% of patients with HFpEF were statistically significant, compared to the hypertensive patients’ group, in which it was present in 31%. Furthermore, the CFR impairment was associated with serum brain natriuretic peptide (BNP) values and HFpEF prognosis [116]. Loffler et al. used CMR stress perfusion to evaluate the microvascular perfusion reserve (MPR) in HFpEF. They found out that CMD was highly present and that MPR was inversely related to ECV [117]. These studies support the hypothesis that HFpEF is a multisystem disease with high endothelial involvement [116,117,118]. 

### 2.3. Heart Failure with Preserved Ejection Fraction and the Inflammation-Metabolic Pathway 

Systemic inflammation is an important pathophysiological mechanism involved in the HFpEF onset and progression. The HFpEF presence and associated conditions have been demonstrated in many systemic inflammation disorders, such as rheumatoid arthritis, systemic lupus erythematosus, psoriasis, inflammatory bowel diseases [119]. Moreover, many cardiovascular comorbidities may induce a systemic inflammatory state and, therefore, HFpEF [119,120]. In this regard, when acting together cardiovascular risk factors amplify myocardial negative effects [119]. 

Epicardial adipose tissue is enhanced in hyperaldosteronism, hypercortisolism and hypothyroidism conditions. The inflammatory-metabolic HFpEF paradigm recognizes the epicardial adipose tissue as a target of systemic inflammation. Epicardial adipose tissue is subjected to several changes, through which it induces many alterations to contiguous tissues, through a paracrine and/or vasocrine effect. The vasocrine route refers to a newly proposed way of intercellular signaling, whereby adipocytes accumulate around arteries and larger arterioles of striated or cardiac muscle and secrete proinflammatory cytokines into the periadventitial space around these vessels. Under inflammation stimuli, cardiac mesenchymal cells are prone to differentiate in adipocytes, promoting the epicardial adipose tissue expansion. In this regard, the epicardial adipose tissue produces and releases several pro-inflammatory molecules through which it may cause CMD, myocardial fibrosis, myocardial electromechanical dysfunction and atherosclerosis progression, in epicardial vessels. The ventricular myocardium involvement is associated with hampered left ventricle filling and diastolic stiffness [119,121], mainly determined by inflammation related titin phosphorylation [122]. The atrial myocardium involvement is associated with fibrosis and atrial fibrillation [119,123,124,125,126]. Inflammatory-metabolic HFpEF is typical phenotype of aged women with many cardiovascular comorbidities. Women have a more developed epicardial adipose tissue and enhanced intramyocardial fat storage [119,127]. In this regard, women have a more impaired ventricle-arterial coupling, worsen arterial stiffness and more accentuate ventricular concentric remodeling than man.

Obesity prevalence in HFpEF patients is about 70% [121] and the epicardial or intramyocardial fat amount may be an indicator of disease severity [128]. CMR is the gold standard for the non-invasive study of myocardial fat [129]. It allows to demonstrate the association between fat and fibrosis, as showed by Wu et al. [130]. Moreover, Wu et al. compared patients with HFpEF and HFrEF, in terms of intramyocardial and epicardial fat values. They found out that the intramyocardial fat was significantly increased in the HFpEF group, while the epicardial type prevailed in the HFrEF group. Diastolic dysfunction imaging parameters showed correlation with intramyocardial fat storage, in female gender, while in the male gender the strongest correlation was with the amount of epicardial fat [127]. According to Mahmod et al. myocardial triglycerides storage and lipotoxicity were significantly more present in the HFpEF patients group than in the control group. Moreover, their presence was independently associated with impaired diastolic strain rate, at CMR feature tracking [131]. 

Epicardial adipose tissue expression and inflammation are associated with circulating hormones levels. In this regard, natriuretic peptides reduce epicardial adipose tissue expansion and inflammation, as well as adipogenesis. Moreover, they counteract myocardial fibrosis and CMD [119,132,133]. On the other hand, leptin promotes aldosterone synthesis, as well as monocytes and T cells activation [119,134,135]. Aldosterone stimulates myocardial fibrosis, CMD and promotes diabetes and myocardial metabolic alterations. In women the leptin and aldosterone effects are more pronounced than man, while obese and post-menopausal women show reduced natriuretic peptides circulating levels. 

From the hemodynamic point of view, inflammatory-metabolic HFpEF is characterized by sodium retention and glomerular resorption, systemic venous capacitance reduction and arterial blood pressure increase. The latter allows the distinction between inflammatory HFpEF from infiltrative and hypertrophic cardiomyopathies [119]. 

Considering the relationship among other HFpEF risk factors and inflammation, RAAS activation induces monocytes migration and M2 macrophages myocardium infiltration, TGF-β production and myocardial fibrosis [44]. Metabolic and energetic state, as well as, ROS production and mitochondrial activity are regulated by several proteins, such as Sirtuins. In this regard, sirtuin is a mitochondrial deacetylase involved in several mitochondrial function, such as ROS and ATP production. Endothelial cells SIRT3 levels are inversely related with aging, diabetes, coronary microvascular rarefaction and endothelial cells reprogramming. Endothelial cells SIRT3 deficiency is associated with ROS production, NO-cGMP pathway, CFR impairment and diastolic dysfunction. Also, hypoxia-related angiogenesis is compromised in case of SIRT3 deficiency. The latter is due to vascular VEGF, Angiopoietin 1 (Ang-1), Hypoxia-inducible factor 1-alpha (HIF-1α) and Hypoxia-inducible factor 2-alpha (HIF-2α) impairment. This condition amplifies the microvascular rarefaction. Moreover, SIRT3 deficiency is also associated with increased Notch 2 and TGF-β1 production that promotes myocardial fibrosis [103]. The lack of SIRT3 is also associated with p53 acetylation. Increased p53 activity induces cell’s senescence and angiogenesis inhibition, which are involved in diastolic dysfunction. Aging is directly related with diastolic dysfunction. Several morphological features which occur with aging may play a role in the diastolic dysfunction determinism, such as myofibril disarray, cardiomyocytes nuclear size increase, cardiomyocytes death and fibroblasts substitution. These changes may be induced by Akt inhibition and p53 activation, which have been observed in human myocardial biopsy sample with diastolic dysfunction. P53 activation is determined by oxidative deoxyribonucleic acid (DNA) damage induced by overload during hypertrophy. Moreover, P53 activation is associated with pathological genes programs, and it is closely related with senescence [103]. Topoisomerase 2 beta has an important role in some gene transcriptional activation process. The topoisomerase 2b knockdown is associated with p53 activation and Akt inhibition, as well as morphological and signaling pathways changes, observed in diastolic dysfunction [136]. 

Van den Hoogen et al. [137] found high plasma Immunoglobulin G1 (IgG1), Immunoglobulin G3 (IgG3) levels and IgG myocardial storage in HFrEF, as well as HFpEF patients. IgG1 and IgG3 stimulate the immune response against cardiac antigens, mainly through complement pathways activation. The inflammatory response induced by the immunoglobulines plays a main role in myocardial remodeling, during HF. In particular, increased IgG1 and IgG3 levels have been observed in end stage HFrEF, as well as in the early phases of HFpEF. Moreover, their circulating values have been related with left ventricular diastolic dysfunction severity, in man. For this reason, IgG1 and IgG3 levels may represent an early marker of HFpEF, that allow to identify this pathological condition, before symptoms onset [137].

Prenner et al. [138] studied the association between serum albumin and HFpEF related adverse outcomes. In HFpEF patients, serum albumin shows an independent and robust prognostic role, in term of death and hospitalization [138]. From the histopathological point of view, reduced serum albumin was associated with myocardial interstitium overexpression, defined by increased myocardial fibrosis detected at autopsies, or by ECV at CMR. Probably, this association may be due to inflammation response, altered nutritional substances intake and liver dysfunction. Moreover, there is a relation between low serum albumin and increased aortic pulsatile wave power, a condition that may contribute to increased aortic and vascular stiffness, as well as CMD [138]. 

Regarding the metabolic aspect, CMR may provide further information in HFpEF. Through the hyperpolarized carbon-13 CMR spectroscopy, an increase in alanine and bicarbonate and no increase in lactate have been demonstrated [139]. Alanine and bicarbonate are pyruvate metabolites, produced by alanine aminotransferase pathway (ALT) and pyruvate dehydrogenase (PDH) pathway, while lactate was produced by lactate dehydrogenase (LDH). In HFpEF heart, a metabolic switch occurs, and energy is no longer produced starting from fatty acids, but through glycolysis [139,140], with a greater PDH activation, compared to healthy controls [139,141]. The increased ALT activity reflects the hypertrophy that involves a greater nucleic acids and amino acids consumption [139,142]. The energetic sources change is an early modification that precedes the structural and functional alterations [143]. The water metabolism is also altered in HFpEF and a study with dynamic contrast-enhanced magnetic resonance under cardiac stress conditions provided further information about metabolic status in HFpEF [144]. This imaging technique allowed to quantify the mean intracellular water lifetime (τi), which is inversely related to the sodium–potassium adenosine triphosphatase (Na^+^/K^+^-ATPase) transporter activity [144,145]. Although under cardiac stress, there was a global decrease in τi and, therefore, an upregulation of Na^+^/K^+^-ATPase activity, the regional analysis showed that this aspect was more evident at the apical level, and subsequently, at the anterior and anterior-lateral wall level. The inferior sectors showed no significant τi decrease. This model traces the metabolic changes in HFpEF and explains why there is much expectation in the use of drugs that act on an energy/metabolic level, such as sodium glucose co-transporter-2 (SGLT-2) inhibitors, in patients with HF and metabolic dysregulation [144,145,146]. 

## 3. Precision Medicine and Personalized Approach to Heart Failure with Preserved Ejection Fraction: The Genetic and Epigenetic Paradigm

The pathophysiology complexity and the lack of specific therapy against HFpEF made this syndrome particularly prone to a personalized and precision medicine approach. The target is to define the HFpEF genetic and molecular basis, in order to predict the pathophysiological and clinical evolution, the patient’s outcome, as well as to find possible innovative therapeutic targets [147]. Hahn et al. [148] identified a specific transcriptome in patients with HFpEF, which differs from healthy controls, as well as HFrEF patients. Patients underwent endomyocardial biopsy from which RNA sequences were obtained to evaluate gene expression and demonstrate the presence of genetic susceptibility independent by HFpEF traditional risk factors. In HFpEF patients, an upregulation of energy production related genes has been observed [148]. It can be associated with the higher prevalence of high BMI, among HFpEF patients, but also with the necessity to perfuse more tissue quantity. Several genes involved in autophagy, angiogenesis and endoplasmic reticulum activity are downregulated in HFpEF. Moreover, different HFpEF trascriptomic subgroups, associated with specific clinical patterns have been identified. In this regard, two HFpEF subgroups have been identified: the former consisted in female patients with upregulated inflammatory pathways and myocardial concentric hypertrophy, while the second subgroup showed molecular, clinical features and mortality, similar to HFrEF [148]. 

The importance of epigenetic has been recognized recently and several studies focus on microRNA profiles that characterize HF subtype or stage. MicroRNA have a main role in the epigenetic modulation of gene expression acting at post-transcriptional moment. The presence of circulating microRNA demonstrate that they are released by cells after death probably acting as paracrine molecules. MicroRNA are involved in the different pathophysiological and histopathological aspects, which characterize HFpEF. Moreover, differences about microRNA targets, between HFrEF and HFpEF, have been observed regarding two pathways involving fatty acids biosynthesis and extracellular matrix receptors expression. The rationale of these observation is sustained by several histopathological findings that characterize HFpEF, as the minor expression of extracellular matrix destruction enzymes, such as metalloproteinase-2, and the enhanced expression of pro-fibrotic markers, such as galectin- 3 and the soluble type of IL-1 receptor like 1 (ST2) [149,150]. Several microRNA are highly expressed in patients with HFpEF such as miR-3908 and miR-3135b or hsa-miR-30a-5p, hsa-miR-181a-2–3p, hsa-miR-199b-5p, hsa-miR-486–5p, hsa-miR-191–5p, hsa-miR-106a-5p, hsa-miR-660–5p, and hsa-miR-193a-5p [149,151]. miR-101a may play a role in the regulation of TGF-β pathway, reducing fibrogenesis [149,152]. In diabetic cardiomyopathy, miR-146a is involved in inflammation and subsequent myocardial fibrosis, mediating cytokines production, through NFκB pathway. miR-155 is associated with diabetes and obesity and it is involved in cardiomyocytes adverse remodeling seen in HF [153]. 

Macrophages are involved in tissue remodeling taking part in inflammatory response. Early phase of HFpEF is characterized by systemic inflammation outspread and the subsequent cardiac inflammation. In this context, macrophages, according with their polarization state, mediate the interaction between inflammatory cells and cardiac cells contributing to the cardiac remodeling [153,154,155]. Macrophages M1 polarization is associated with pro-inflammatory activity and cell death, while macrophages M2 polarization is associated with fibrogenesis, tissue repair and immunosuppression [154]. Many microRNA such as miR-125b, miR-127, miR-9 and miR-155 are involved in myocardial macrophage M1 polarization, while miR-223, miR-124, miR-132, miR-125a-5p, miR-34a and miR-146a are involved in myocardial macrophage M2 polarization [153,154,155]. 

Other microRNAs are involved in proteins post-translational modification such as the ubiquitin induced proteolysis regulation [149]. miR-126 is the most represented endothelial microRNA and its expression is markedly reduced in case of endothelial dysfunction. The lack of miR-126 is associated with several microvascular abnormalities seen in HFpEF, such as the loss of vascular layer integrity, microvascular inflammation and hemorrhage [153,156]. Transcoronary gradient represents the difference of microRNA concentration across the coronary circulation, between coronary sinus and coronary arterial district and it is a marker of mircoRNA release within coronary circulation. Elevated miR-92a and -133 transcoronary gradient is associated with CMD [153,157] while miR-138 may represent a target to restore endothelial function and NO production, in HFpEF. In HFpEF, microRNAs are involved also in cardiomyocytes structural alterations and extracellular matrix composition regulation. In this regard, miR-17–92 cluster is crucial in extracellular matrix genes expression associated with aging [153,158]. miR-22 is the most represented heart microRNA. Cardiomyocytes miR-22 expression is increased under angiotensin II stimulation. It regulates the sarcoplasmic reticulum reuptake modulating SERCA2 activity [153,159]. miR-22 is markedly associated with cardiomyocytes hypertrophy and cardiac fibrosis [153,160]. MiR-21, a pro-fibrotic microRNA, regulates Extracellular Signal-Regulated Kinase Mitogen-Activated Protein (ERK-MAP) kinase pathway, involved in fibroblasts vitality, as well as TGF-β pathway. MiR-208b is associated with titin structural modification and dysfunction. miR-1 regulates calmodulin activity, in smooth muscle cells during HF [153,161]. MiR-181b regulates PKG-1 expression and it represents a marker of cardiomyocytes hypertrophy [152]. 

Gender differences in HFpEF patients have been observed in term of microRNA expression. Florijn et al. demonstrated that plasma miR-224 and miR-452 are predominantly expressed by HFpEF diabetic women, instead of diabetic HFpEF man. Moreover, miR-34a is associated with kidney disease, diabetes mellitus and gender in patients with diastolic dysfunction, while decreased miR-34a, -224 and -452 plasma levels have been observed in diabetic patients with diastolic dysfunction and diabetic women with glomerular filtration rate under 60 mL/min [162]. The definition of genetic and epigenetic alterations, in particular microRNA role, is a key point in the setting of a personalized approach for the diagnosis and management of HFpEF and HF in general.

## 4. Role of Myocardial Tissue Characterization and Pathophysiological Mechanisms for the Identification of New Therapeutic Targets 

### 4.1. The Renin-Angiotensin-Aldosterone System and Neprilysin Pathway

Angiotensin II receptor type 1 (AT1) stimulation induces myocardial hypertrophy and fibrosis participating to HF worsening [163]. RAAS inhibitors reduce HFrEF related morbidity and mortality, while their role in HFpEF is controversial [164,165,166]. Over the past years multiple clinical studies failed to demonstrate a direct outcome improvement in HFpEF treated with RAAS inhibitors [167,168,169,170,171], while the positive role of β-blockers [172], angiotensin receptor blockers (ARBs), and mineralocorticoid receptor antagonists (MRAs) [173], on mortality and morbidity reduction, in HFrEF patients has been demonstrated. Taking into account the positive role of RAAS blockade in hypertensive patients, as well as the hypertension prevalence in HFpEF subjects, RAAS inhibitors would be expected to improve clinical outcomes in those patients. However, many trials evaluating RAAS blockade in HFpEF patients were often ambivalent. The Irbesartan in Heart Failure With Preserved Ejection Fraction (I-PRESERVE) study investigated the ARB irbesartan effect versus placebo, in patients with HFpEF [174]. This study did not show a significant reduction in all-cause death and in hospitalization due to cardiovascular disease, in patients with HFpEF taking Irbesartan. However, this study had several limitations. First, HFpEF diagnosis was often challenging. Secondly, the adopted dose of Irbesartan (300 mg/die) for the study may not have been the optimal one to treat HFpEF and furthermore, the 34% of study population did not take therapy continuously [174]. However, Lund et al. [175] reported a significant treatment benefit in HF with mid-range ejection fraction (HFmrEF), in a post hoc analysis. The Valsartan In Diastolic Dysfunction (VALIDD) study compared valsartan to other antihypertensive drugs in patients with diastolic dysfunction and hypertension [176]. In both groups, diastolic function improved after reduction of blood pressure, notwithstanding the antihypertensive treatment. 

A revolution of HFrEF treatment was carried out by the angiotensin receptor neprilysin inhibitor LCZ696, that combine the two acting molecules, valsartan and sacubitril. By inhibition of neprilysin, sacubitril increases atrial natriuretic peptide (ANP), BNP and C-type natriuretic peptide (CNP) plasma levels [177], through the guanylyl cyclase activation and cGMP synthesis. Moreover, natriuretic peptides prevent myocardial fibrosis and help to low blood pressure, due to vasodilation and increased diuresis. The Prospective Comparison of angiotensin receptor neprilysin inhibitor (ARNI) with ARB Global Outcomes in HF With Preserved Ejection Fraction (PARAGON-HF) trial about the ARNI use in patients with HFpEF did not show a statistically significant lower rate of hospitalization for HF and death from cardiovascular causes. However, it suggested possible benefit among woman and patients with a left ventricular ejection fraction between 45–57% [178]. In a recent network meta-analysis, Kuno et al. [179] compared outcomes of different RAAS antagonists with each other and with placebo, in HFpEF patients. The combination of sacubitril-valsartan is associated with lower HF hospitalizations, but not lower mortality rate, in those patients. No statistical difference in all-cause mortality and cardiovascular mortality, among ACE-I, ARBs, MRA, ARNI, and placebo has been demonstrated. Mineralocorticoid receptor antagonists (MRAs) prevent aldosterone’s effect on myocardial fibrosis [180]. In the Effect of Spironolactone on Diastolic Function and Exercise Capacity in Patients With Heart Failure With Preserved Ejection Fraction (ALDO-DHF) trial, spironolactone showed a positive effect on diastolic function through the E/e′ ratio reduction [181]. It decreased left ventricular hypertrophy and N-terminal pro-B-type natriuretic peptide (NT-proBNP) levels. Despite its role on quantitative markers, HF symptoms, exercise tolerance, and life quality have not been significantly affected by spironolactone. In the Treatment of Preserved Cardiac Function Heart Failure With an Aldosterone Antagonist (TOPCAT) trial the MRA spironolactone was added to medical therapy with β-blockers and angiotensin converting enzyme (ACE) inhibitors, in HFpEF patients [182]. In theTOPCAT trial, Spironolactone addition did not determine a significant reduction in the primary composite of cardiovascular death, hospitalization for HF and aborted cardiac arrest [182].

### 4.2. The Oxidative Stress and the Nitric Oxide Pathway 

HF is associated with oxidative stress, which affects myocardium and vasculature. Recent evidence suggests that oxidative stress may be the link between obesity, diabetes mellitus, and related complications. In obese patients, there is an increasing level of reactive oxygen and nitrogen species that directly correlate with central adiposity. 

The cGMP pathway plays a fundamental role in regulating normal cardiovascular function and its lack in HFpEF subjects leads to endothelial dysfunction. Endothelial dysfunction is related to cGMP deficiency that is caused by insufficient stimulation of soluble guanylate cyclase (sGC) and impaired NO bioavailability. This promotes CMD, myocardial and vascular stiffness [183].

Correction of myocardial PKG activity and cGMP pathway has been proposed as a target for specific HFpEF treatment, but Sildenafil related phosphodiesterase type 5 (PDE5) inhibition did not show difference compared to placebo, in HFpEF patients [184].

The sGC stimulator Vericiguat was studied in the SOluble guanylate Cyclase stimulatoR in heArT failurE Studies (SOCRATES) programme [185]. In the HFpEF group, it did not show the achievement of primary end points of NT-proBNP or left atrial volume, over a 12-week treatment period. However, an exploratory post hoc analysis showed clinically significant improvements in health status defined by Kansas City Cardiomyopathy Questionnaire [186].

Considering the hypothesis of decreased NO availability in HFpEF, nitrates have been proposed to restore NO balance, in order to improve endothelial-myocyte paracrine signaling. The Nitrate’s Effect on Activity Tolerance (NEAT-HFpEF) trial was conducted to investigate the role of isosorbide mononitrates in HFpEF management [187]. Isosorbide mononitrate did not improve the daily activity level, exercise capacity, quality of life or NT-proBNP levels in patients with HFpEF. This is in contrast with nitrates’ positive effect in HFrEF subjects and may be related to the pathophysiologic differences between the two HF types. Common HFpEF features, such as increased ventricular and vascular stiffness, chronotropic incompetence and altered baroreflex sensitivity may limit the hemodynamic benefits of nitrates, in HFpEF patients. 

The inorganic nitrate pathway represents a different way to restore NO signal [188]. Unlike the organic nitrates, inorganic nitrite is converted to NO, in presence of hypoxia and acidosis, which develop in tissues and venous circulation during exercise. Moreover, there is no tolerance to nitrite. For this reason, inorganic nitrate/nitrite has been proposed as a target therapy because NO is crucial at the time of greatest need, such as during exercise. Notwithstanding this promising assumption, results of different trials such as the Inorganic Nitrite Delivery to Improve Exercise Capacity in Heart Failure With Preserved Ejection Fraction (INDIE-HFpEF) [188] and the Effect of KNO3 Compared to KCl on Oxygen UpTake in Heart Failure With Preserved Ejection Fraction (KNO3CKOUT) (NCT02840799) are ambivalent. 

A new class of antioxidant peptides named “Szeto-Schiller peptides (SS peptides)” is under investigation in HF. SS peptides belong to a class of antioxidant that bind to cardiolipin, an important phospholipid of the inner mitochondrial membrane. These peptides protect cardiolipin from oxidation and reduce mitochondrial oxidative damage. One of the most prominent of these peptides is elamipretide (MTP-131, SS31). In patients with HFpEF, elamipretide reduced left-ventricular end-diastolic volumes, compared to placebo [189]. 

### 4.3. Role of Inflammation, Fibrosis and Calcium Handling as Therapeutic Targets

Proinflammatory cytokines, such as IL-1 are upregulated in HFpEF. They have an important role in the myocardial function impairment. In HFpEF, IL-1 inhibits L-type calcium channels, downregulates phospholamban activity and causes post-transcriptional changes in SERCA2a [190]. Calcium handling dysregulation leads to impaired cardiac relaxation and diastolic dysfunction. The Diastolic Heart Failure-Anakinra Response Trial (D-HART) investigates Anakinra role in patients with diastolic dysfunction [191]. Anakinra is a recombinant IL-1 receptor antagonist, and it decreases inflammatory markers levels, improving the HFpEF patients aerobic exercise capacity. It is still unclear the role of canakinumab, a monoclonal antibody specifically targeting the IL-1β isoform, in HF subjects; in a sub-analysis of the large Canakinumab Antiinflammatory Thrombosis Outcome Study (CANTOS), that include patients with previous myocardial infarction and increased high sensitivity C-reactive proteins levels, canakinumab showed clinical benefit. It remains unclear its role upon diastolic function [192]. 

Myocardial inflammation and fibrosis are target of cell therapy. In animal models Pirfenidone, antifibrogenic drug which target TGF-β signaling, inhibits left ventricular fibrosis and diastolic impairment [193]. Its role in HFpEF subjects is under investigation by the Pirfenidone in Heart Failure with Preserved Ejection Fraction-Rationale and Design (PIROUETTE) trial (NCT02932566).

CD34 is a receptor expressed by bone marrow multipotent progenitor cells, which are reduced in patients with both HFrEF and HFpEF. CD34^+^ cells may be suitable for cell therapy to improve diastolic function in HFpEF. A pilot study with HFpEF subjects showed that treatment with CD34^+^ cells determined diastolic function improvement and NT- proBNP levels decrease [194]. The role of CD34^+^ cell therapy in patients with HFpEF is currently under definition. Dysfunctional calcium handling in HFpEF causes impaired myocardial relaxation. Late inward sodium current (I_Na_) is increased in HF, causing cardiomyocytes calcium overload. Ranolazine inhibits persistent or late I_Na_ in heart muscle [195]. In the Ranolazine for the Treatment of Diastolic Heart Failure (RALI-DHF) study, Ranolazine decreased left ventricular end diastolic pressure, without changing exercise tolerance [196]. As reported by European Society of Cardiology guidelines, Levosimendan, a calcium sensitizer and phosphodiesterase-3 (PDE3) inhibitor with vasodilative properties, can be considered in patients with acute HF and severe reduction of cardiac output [197,198]. It improved diastolic function and right-ventricular systolic function, in patients with advanced HF. Moreover, Levosimendan a role in the inflammatory status regulation, through the IL-6/IL-10 ratio alteration [199]. In this regard, the Hemodynamic Evaluation of Levosimendan in Patients With PH-HFpEF (HELP) trial (NCT03541603) is investigating the effects of Levosimendan in HFpEF patients with left heart disease related pulmonary hypertension.

### 4.4. The Heart Rate and Volemic Status Regulation as Therapeutic Targets

In HFpEF patients, high heart rate predicts poor outcome at sinus rhythm, instead there are no clear correlations between worse prognosis and atrial fibrillation. This finding was confirmed in The Irbesartan in Heart Failure With Preserved Systolic Function (I-PRESERVE) trial [200] and the Meta-analysis Global Group in Chronic Heart Failure (MAGICC) registry [201]. A slowdown in resting heart rate causes a raise in filling pressures [202]. Ivabradine, an inhibitor of the funny current, reduces heart rate and improves vascular stiffness, as well as systolic and diastolic function [203]. In The prEserveD left ventricular ejectIon fraction chronic heart Failure with ivabradine studY (EDIFY) study, ivabradine reduced heart rate, but worsened E/e′ ratio, exercise tolerance, and NT-proBNP levels, in HFpEF patients [204]. Ivabradine heart rate lowering does not provide any consistent benefit, because diastole prolongation does not seem to improve diastolic function and prognosis in patients with HFpEF.

Sodium-Glucose Cotransporter 2 Inhibitors (SGLT2i) showed a striking reduction of cardiovascular events, in patients with T2DM. SGLT2i reduces renal glucose reabsorption, rise urinary glucose excretion and increases diuresis [205,206,207]. In the The Empagliflozin Cardiovascular Outcome Event Trial in Type 2 Diabetes Mellitus Patients Removing Excess Glucose (EMPA-REG OUTCOME) study, empagliflozin leads to a striking reduction of cardiovascular events in high cardiovascular risk T2DM patients. Several aspects, such as the pre-load reduction and the cardiac energetics improvement, through an increase in ketones’ supply, should be involved in the SGLT2i positive effects on cardiovascular and renal outcomes [197,208]. Empagliflozin showed a direct effect on diastolic function improvement in HF [209]. The ongoing EMPagliflozin outcomE tRial in Patients With chrOnic heaRt Failure With Preserved Ejection Fraction (EMPEROR-PRESERVED) and the Dapagliflozin Evaluation to Improve the LIVEs of Patients With PReserved Ejection Fraction Heart Failure (DELIVER) (NCT03619213) trials are respectively studying the empagliflozin and dapaglifozin’s effects on HF hospitalization and cardiovascular mortality reduction, in HFpEF subjects with and without diabetes.

Table 1 contains a summary of the main trials regarding HFpEF therapy according to the targeted pathophysiological pathway.

## 5. Conclusions

**Figure 2:**
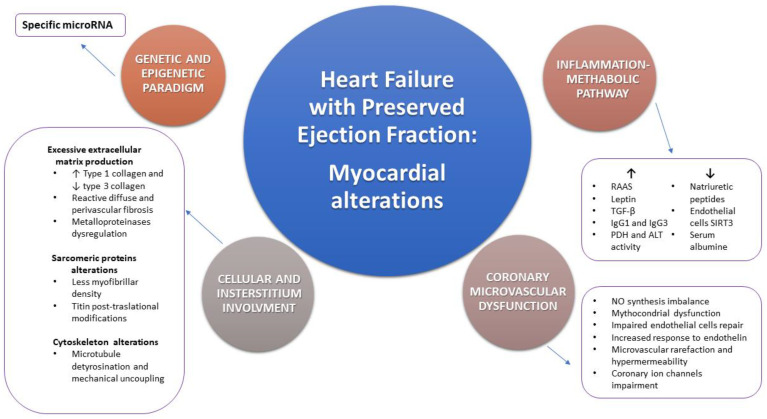
Schematic representation of the main myocardial and coronary histopathological and pathophysiological alterations observed in heart failure with preserved ejection fraction (HFpEF). Cellular and interstitial involvement, coronary microvascular dysfunction, genetic and epigenetic imbalance and the inflammation-metabolic pathway are the main substrates leading to HFpEF. Each of the listed mechanism implies many molecular and ultrastructural alterations. *RAAS: renin angiotensin aldosterone system; TGF-β: transforming growth factor beta; IgG1: immunoglobulin G1; IgG3: immunoglobulin G3; PDH: pyruvate dehydrogenase; ALT: alanine aminotransferase; SIRT3: sirtuin-3; NO: nitric oxide*.

HFpEF is a multifaceted and complex syndrome associated with global high mortality and morbidity rates and its prevalence is constantly increasing. Although it is well known the association among HFpEF and traditional cardiovascular risk factors, myocardial alterations and pathophysiological basis are not well defined yet. In fact, the definition of HFpEF includes a wide spectrum of different myocardial structural alterations. Myocardial hypertrophy and fibrosis, CMD, oxidative stress and inflammation are only some of the main pathological detectable processes at myocardium levels and their *consecutio* in HFpEF onset and progression is not well established. A comprehensive overview of mechanisms involved in myocardial alterations observed in HFpEF are summarized in Figure 2. Only the accurate and detailed characterization of myocardial tissue allows the full comprehension of its pathophysiological mechanisms together with the possibility to identify new therapeutic targets, in order to specifically treat HFpEF, beyond traditional cardiovascular risk factors control. Myocardial tissue characterization is certainly possible through endomyocardial biopsy and histological analysis. However, its invasiveness hampers the possibility to use it on large scale population. Actually, the improvement of imaging techniques, such as CMR and their greater diffusion in clinical practice allows myocardial tissue characterization in a non-invasive way. Moreover, CMR allows a dynamic study of myocardial metabolism, both during stress and at rest, in HFpEF patients. The reliable use of imaging techniques to characterize myocardial tissue in HFpEF may promote an earlier, non-invasive and large-scale diagnosis, and this could allow to identify this syndrome during its earlier phase of development, when it is more likely to be successfully treated. However, the correlation between myocardial histopathological findings and imaging aspects is still a challenge for medicine and further evidence is needed. 

## Figures and Tables

**Table 1:** Main clinical trials of pharmacological therapy in heart failure with preserved ejection fraction.

Trial Name (Years)	Drug (Posology)	Sample Size	ClinicalTrials.gov Identifier	Follow up Duration	Results
RAAS and Neprylisin Pathway					
I-PRESERVE (2002–2008)	Irbesartan (Oral, from 75 to 300 mg daily vs. placebo)	4128	NCT00095238	49.5 months	Irbesartan did not improve outcomes (death from any cause or hospitalization for CV cause)
CHARM-PRESERVED (1999–2003)	Candesartan(32 mg once daily vs. placebo)	3023	NCT00634712	36.6 months	Candesartan did not improve outcomes (cardiovascular mortality or hospitalization due to congestive HF)
TOPCAT(2006–2013)	Spironolactone(Oral, 15 mg to 45 mg daily vs. placebo)	3445	NCT00094302	39 months	Spironolactone did not significantly reduce the incidence of the primary composite outcome of death from CV causes, aborted cardiac arrest, or hospitalization for the HF management
ALDO-DHF (2007–2012)	Spironolactone(Oral, 25 mg daily vs. placebo)	422	ISRCTN94726526	12 months	Long-term aldosterone receptor blockade improved left ventricular diastolic function but did not affect maximal exercise capacity, symptoms or quality of life
PARAGON-HF (2019–2019)	Sacubitril/Valsartan(Oral. Two periods:(1) a single-blind treatment from 3 to 8 weeks with valsartan 80 mg bid, followed by sacubitril/valsartan 100 mg bid(2) a double-blind randomized treatment with sacubitril/valsartan 200 mg bid or valsartan 160 mg bid	4822	NCT01920711	35 months	Sacubitril–valsartan did not result in a significantly lower rate of total hospitalizations for HF and death from CV causes
**Oxidative stress and Nitric oxide pathway**					
KNO3CK OUT-HFpEF (2016–2022)	Potassium Nitrate (KNO3)(Oral, 6 millimoles of inorganic nitrate per capsule, three times daily for 6 weeks vs. placebo)	76	NCT02840799	N/A	Outcome: VO_2_ (ongoing study)
INDIE-HFpEF (2016–2018)	Inorganic nitrite or nitrate preparations(Nebulized sodium nitrite at 46 mg then 80 mg three times per day vs. placebo)	105	NCT02742129	17 months	Administration of inhaled inorganic nitrite for 4 weeks, compared with placebo, did not result in significant improvement in exercise capacity and VO_2_
SOCRATES-PRESERVED (2013–2015)	Vericiguat(Oral, 2.5 mg once daily for 2 weeks, up-titration to 5 mg orally once daily for 2 weeks, up-titration to 10 mg orally once daily for 8 weeks vs. placebo)	477	NCT01951638	16 weeks	Vericiguat, did not change NT-proBNP levels at 12 weeks compared with placebo but it was associated with improvements in quality of life
NEAT-HFpEF (2014–2016)	Isosorbide mononitrate(6-week dose-escalation regimen of isosorbide mononitrate, from 30 mg to 60 mg to 120 mg once daily vs. placebo)	110	NCT02053493	6 weeks	Patients who received isosorbide mononitrate were less active and did not have better quality of life or submaximal exercise capacity than patients who received placebo
**Inflammation pathway and calcium handling**					
D-HART (2014–2017)	Anakinra(Subcutaneous, Interleukin-1 blockade, 100 mg subcutaneously once daily for 12 weeks vs. placebo)	60	NCT02173548	12 weeks	Anakinra significantly reduced the systemic inflammatory response and improved the aerobic exercise capacity of patients with HFpEF and elevated plasma CRP levels.
HELP (2018–2020)	Levosimendan(Injectable Solution 0.075–0.1 µg/kg/min for 24 h weekly vs. placebo)	38	NCT03541603	6 weeks	Levosimendan infusion did not affect exercise-PCWP but did reduce PCWP incorporating data from rest and exercise, in tandem with increased 6 min-walking-test
**Fibrosis pathway**					
PIROUETTE (2017–2020)	Pirfenidone(Oral, 801 mg three times daily vs. placebo	129	NCT02932566	12 months	Change in myocardial ECV from baseline to 52 weeks
**SGLT-2 inhibition**					
EMPEROR-Preserved (2017–2021)	Empagliflozin(Oral, 10 mg daily vs. placebo)	5988	NCT03057951	20 months	Time to first event of adjudicated CV death or HHF (ongoing)
DELIVER (2018–2022)	Dapagliflozin (Oral, 10 mg daily vs. placebo)	6263	NCT03619213	27 months	Composite of CV death, HHF and urgent HF visit (ongoing)

RAAS: renin angiotensin aldosterone system; CV: cardiovascular; HF: heart failure; VO_2_: maximal oxygen consumption; NT-proBNP: N-terminal-pro hormone brain natriuretic peptide; PCWP: pulmonary capillary wedge pressure; HHF: heart failure hospitalization; CRP: C-reactive protein; ECV: extracellular volume fraction.
